# Using targeted therapy to promote a pro-inflammatory tumour microenvironment and anti-tumour immune response in high grade serous ovarian cancer

**DOI:** 10.1038/s41416-026-03416-y

**Published:** 2026-04-07

**Authors:** Zhen Zeng, Anastasia Gandini, Rituparna Bhatt, Martina Proctor, Nicole-Lisa Li-Ann Goh, Shivam Vora, Thomas P. Walsh, Sherry Y. Wu, Kaltin Ferguson, Jermaine I. Coward, Snehlata Kumari, Nikolas K. Haass, James W. Wells, Janet Hardy, Lewis Perrin, Yaowu He, John D. Hooper, Gwo-Yaw Ho, Jazmina L. Gonzalez Cruz, Brian Gabrielli

**Affiliations:** 1 Mater Research Institute, The University of Queensland, Brisbane, QLD Australia; 2 Faculty of Health, Medicine and Behavioral Sciences, School of Biomedical Sciences, The University of Queensland, Brisbane, QLD Australia; 3 Icon Cancer Centre, and Faculty of Medicine, University of Queensland, Brisbane, QLD Australia; 4 Frazer Institute, Faculty of Health, Medicine and Behavioral Sciences, The University of Queensland, Brisbane, QLD Australia; 5 Monash University Department of Medicine, School of Clinical Sciences, Melbourne, VIC Australia

**Keywords:** Targeted therapies, Ovarian cancer, Cancer immunotherapy

## Abstract

**Background:**

High-grade serous ovarian cancer (HGSOC) is characterized by elevated replication stress and an immunosuppressive microenvironment. A synergistic combination of checkpoint kinase 1 inhibitor (CHK1i) with low-dose hydroxyurea (LDHU) promotes a unique ATR-independent moderate replication stress response with potent anti-tumour effects. The ability of this approach to reprogram the tumour immune microenvironment (TIME) to overcome the immunosuppression and promote an anti-tumour immune response in HGSOC is the focus of this study.

**Methods:**

We investigated the therapeutic potential of CHK1i+LDHU in established HGSOC cell cultures, fresh tumour cell explants from HGSOC patient ascites, and syngeneic mouse models, assessing tumour cell killing, immunogenic cell death, pro-inflammatory cytokine/chemokine expression, and anti-tumour immune responses.

**Results:**

CHK1i+LDHU effectively killed ovarian cancer cells regardless of prior chemotherapy responses, *BRCA2* mutation and homologous recombination repair status in vitro. In vivo, treatment significantly reduced tumour burden and ascites accumulation. CHK1i+LDHU enhanced expression of pro-inflammatory cytokines/chemokines and triggered immunogenic cell death in tumour. In syngeneic models, treatment promoted CD8^+^ cytotoxic T cell-dependent anti-tumour responses and reduced immunosuppressive signalling within the TIME.

**Conclusions:**

CHK1i+LDHU is a promising therapy for chemotherapy-resistant HGSOC, combining direct cytotoxic effects with reprogramming the TIME to reduce immunosuppression and activate a CD8^+^ T cell-dependent anti-tumour response.

## Introduction

High grade serous ovarian cancer (HGSOC) is the most lethal gynaecologic malignancy, with a 5-year survival rate of 35-40% [1]. The current standard of care involves platinum-based chemotherapy, often combined with taxanes. While initially effective, over 70% of patients experience relapse within 2 years [2]. Chemotherapy is associated with high-grade toxicities that severely impact patient quality of life and, importantly, contributes to immunosuppression [3]. Relapse-free survival is extended in patients with homologous recombination repair defective (HRD) tumours ( < 50% of patients) treated with poly-ADP ribose polymerase inhibitors (PARPi) [2, 4]. However, this benefit is limited in non-BRCA mutant ovarian cancer, and emerging data indicate increased toxicity associated with PARPi use in the relapse setting [5]. Immunotherapy response rates are <20% [6], underscoring the profound immunosuppressive barriers in HGSOC [7]. This low response rate is particularly surprising given that a high proportion of HGSOC cases exhibit tumour-infiltrating lymphocytes, and CD8^+^ T cell levels are prognostic for survival [6]. This indicates the tumour immune microenvironment (TIME) is highly immunosuppressive, severely limiting the efficacy of immunotherapies [8]. Elevated levels of regulatory T cells (Tregs), M2 type macrophages and myeloid derived suppressor cells (MDSCs) are commonly observed in HGSOC, contributing to this immunosuppressive TIME [9]. Consequently, once patients develop resistance to chemotherapy, treatment options are severely limited. There is an urgent need for targeted therapies that not only selectively treat chemo-resistant HCSOC but also modulate the TIME to overcome the immunosuppression and promote robust anti-tumour immune responses.

Checkpoint kinase 1 inhibitors (CHK1i) effectively target cells with elevated replication stress [10–12], a common feature of ovarian cancer [13] and has been identified as an early feature of the disease [14]. CHK1i have been shown to be effective in PARP inhibitor resistant HGSOC preclinical models [12]. The CHK1i prexasertib achieved a 30% disease control rate but and overall response rate of 10% in a phase II trial in platinum refractory HGSOC patients [15, 16]. Prexesertib combination with the PARPi olaparib demonstrated effectiveness preclinically and in a phase I trial in PARPi resistant patients but was associated with significant haematological toxicity [17–19]. Replication stress inducers such as gemcitabine combine synergistically with CHK1i [20–22]. Clinical trials of these combinations across various cancer types ( > 12 trials), many in the recurrent, relapsed, or refractory setting [23], demonstrated anti-tumour efficacy but also revealed frequent severe haematological toxicities [24, 25]. These toxicities arise from CHK1i chemo-sensitising all tissue to the replication stress inducers which promote high-level replication stress in both tumour and normal tissue [26].

Subclinical doses of hydroxyurea (low-dose HU; LDHU) that have little effect on tumour growth either in vitro or in vivo [10] synergise strongly with CHK1i in vitro and in vivo in other cancer types [10, 27]. Unlike clinically used replication stress inducers such as gemcitabine that trigger an ATR/CHK1-dependent S phase checkpoint arrest [28], LDHU triggers a unique CHK1-dependent reorganisation of replication origin firing [29]. LDHU selectively sensitises tumours with elevated endogenous replication stress to CHK1i [10, 30]. CHK1i are unlikely to be clinically viable as single agents, while most of the combinations trialled to date have excessive toxicity. This makes the combination of CHK1i+LDHU an attractive option owing to its efficacy, low normal tissue toxicity in vivo and its ability to maintain robust adaptive immune response [10, 31].

In this study, we have investigated whether low dose hydroxyurea elicits the same synergy with the selective CHK1i SRA737 [32] at a dose ~20% of its reported mean maximum plasma concentration (Cmax) in HGSOC as reported in other cancer types [10, 30]. Using a cross section of different molecular subtypes and treatment resistant HGSOC models, we assessed whether the broad tumour-killing efficacy observed elsewhere extends to HGSOC. Importantly, given the highly immunosuppressive TIME in HGSOC – which underlies its poor response to current immunotherapies – we also examined whether this combination can not only spare immune function but actively trigger anti-tumour immune responses. Part of this response is likely to be tumour expressed cytokines and chemokines for example Ccl2, Cxcl10, Tnf, that promote an inflammatory state within the tumour microenvironment that could recruit anti-tumour immune cells into the TIME [33]. Specifically, in HGSOC, we investigated whether tumour cell death induced by CHK1i+LDHU is immunogenic, whether it remodels the cytokine and chemokine milieu to reprogram the TIME, and whether it overcomes key immunosuppressive barriers. We further identified the critical immune cell population mediating the treatment related anti-tumour effect. We demonstrate that CHK1i+LDHU efficiently kills a broad range of genotypes and chemo-resistant ovarian cancer cells. In vivo, this combination significantly reduced tumour burden and ascites accumulation by inducing immunogenic tumour cell death and activating a CD8^+^ T cell-dependent immune response, accompanied by a favourable reprogramming of the TIME. Together, these finding highlight CHK1i+LDHU as a promising therapeutic strategy for HGSOC, with the potential to overcome its profound immunosuppressive barriers and stimulate effective anti-tumour immunity.

## Methods

### Cell lines

Luciferase-tagged mouse ovarian cancer cell lines ID8-p53^WT^ and ID8-p53^−/−^ [34, 35], human ovarian cancer cell lines OVCA420 (RRID:CVCL_3935), PEO1 (RRID:CVCL_2686), PEO4 (RRID:CVCL_2690), FUOV-1 (RRID:CVCL_2047), OVCAR3 (RRID:CVCL_0465), OVCAR8 (RRID:CVCL_1629) and Kuramochi (RRID:CVCL_1345) were derived as described in Supplementary Table S1. A panel of four cell lines, PEO1, OVCAR8, FUOV-1 and Kuramochi representing primary and metastatic deposits, BRCA wild type and mutant, chemo naive and derived from patients who have become chemo-resistant were used in all experiments. In order to better reflect the genetic diversity expected in a normal patient population, other cell lines were added to this panel to further increase this diversity. A panel of three HGOSC patient ascites derived cell lines were derived as described in the Supplementary Material. Cells were cultured in high glucose DMEM (Gibco) with 10% heat treated fetal bovine serum (FBS; Bovogen), 1 mM Sodium pyruvate (Gibco), GlutaMAX (Gibco), 20 mM HEPES (Sigma-Aldrich) and x1 antibiotic-antimycotic (Gibco). Cell cultures were maintained in a Binder low oxygen incubator at 37 °C, 5% CO_2_ and 2% O_2_ and were tested mycoplasma free.

### Dose-response assay

All cell lines were seeded in 96-well plates and treated with increasing concentrations of CHK1i (SRA737, Sierra Oncology) with or without combination with 0.2 mM HU (Sigma). Cells were assessed for viability after treatment for 3 days using resazurin (Sigma). PEO4 was assessed after 6 days dues to its longer doubling time of 48 h compared to the other cell lines (24–30 h) (Ref).

### Time-lapse cell viability killing assay

Indicated cells (500-3000 in 100ul media per well) were seeded into 96-well plates and treated with or without 1 µM CHK1i (SRA737), ATR inhibitor 2 μM VE-821 (SelleckChem) and 0.2 (low dose) or 2 mM (high dose) hydroxyurea. Sytox Green (125 nM; Molecular Probes) to mark dead cells was also added and cells were imaged every 4 h at 10× magnification for up to 3 days using an IncuCyte S3 live cell imaging system (RRID:SCR_023147). Analysis was performed using IncuCyte software (RRID:SCR_025367) to identify total number of cells, and percentage of cells positive for Sytox Green staining for each frame. Data was presented as total cell counts for proliferation, percent cell death from the number of Sytox stained cells/ total cell number for each time point, and percent viable cells.

### Immunoblotting

Ovarian cancer cell lines were treated with or without 1 µM CHK1i (SRA737) and 0.2 mM hydroxyurea for 24 h. Cell pellets were lysed and analysed by immunoblotting, using antibodies against RPA2, γH2AX and Alpha-Tubulin (Supplementary Table S3). Antibodies were diluted 1:1000. Proteins were visualized using chemiluminescence (Fusion SL Viber Lourmat).

### RT-qPCR

Murine (ID8-p53^WT^ and ID8-p53^−/−^) and human (PEO1, PEO4, OVCAR8, OVCA420, FUOV-1 and Kuramochi) ovarian cancer cell lines were treated without or with CHK1i combination (1 µM CHK1i and 0.2 mM hydroxyurea) for 24 h. Total RNA was extracted using TRIzol (Invitrogen) per the manufacturer’s protocol. RNA concentration and purity were assessed using a NanoDrop One/OneC. cDNA synthesis was performed with 2 µg of RNA using the High-Capacity cDNA Reverse Transcription Kit (Applied Biosystems: 4368814), followed by a 1:10 dilution with nuclease-free water. Quantitative PCR was conducted using PowerUp SYBR Green Master Mix (Applied Biosystems) on a ViiA 7 Real-Time PCR System or QuantStudio 7 Flex Real-Time PCR System. Custom and predesigned KiCqStart Primers (MERK) were employed for qPCR (Supplementary Table S4). The genes analysed are listed in Table S3. Fold change in gene expression was calculated using 2^–(ΔCt), where ΔCt represents the difference between the average Ct of the target gene and the reference gene, actin (mouse) or YWHAZ (human) [36].

### Cytokine bead array

LEGENDplex HU Essential Immune Response Panel (BioLegend, San Diego, CA, USA) assayed six of the cytokines/chemokines assayed in the qRT-PCR panel above (CCL5 was not part of this panel) was used according to the manufacturer’s instructions to assess the cytokines present in cell supernatants after 48 h CHK1i+LDHU treatment. Samples were performed in duplicate and analysed on a CytoFLEX S Flow Cytometer, RRID:SCR_019627 (Beckman Coulter, Lane Cove, Australia). Acquired data were analysed using provided LEGENDplex Data Analysis Software. Budget constraints did not allow for assessment of the murine cytokine/chemokine secretion.

### ICD marker

Human ovarian cancer cell lines were cultured for 24 h in the presence of DMSO or CHK1i +LDHU, then harvested and stained for surface expression with anti-calreticulin-AF647, anti-HSP90-PE or relevant isotype controls (diluted in 1:100). The delta mean-fluorescence-intensity (ΔMFI) values were calculated by taking the mean values on live cells. Stained cells were analysed using an LSR-Fortessa X20 Flow Cytometer (BD BioSciences) with FACSDiva software, RRID:SCR_001456 (Becton Dickinson, Sparks, MD, USA). Acquired data were analysed using FlowJo software, RRID:SCR_008520 (TreeStar Inc., Ashland, OR, USA).

### ICD assay

C57BL/6 J mice were immunised with ID8-p53^−/−^ cells treated in vitro 1 µM CHK1i (SRA737) + 0.2 mM HU for 24 h. Freeze-thaw killed ID8-p53^−/−^ cells were used as a negative control for ICD. At 9 days after immunisation mice were rechallenged with live ID8-p53^−/−^ cells into the opposite flank and tumour burden was monitored by IVIS in vivo using 5 mice each treatment.

### Mouse tumour assays

Experiments were performed with approval from The University of Queensland Animal Ethics Committee (2021/AE000249). Luciferase-labelled ID8-p53^WT^ and ID8-p53^−/−^ cells (1.5 × 10^6^) in 200 μl of Hank’s balanced salt solution were intraperitoneally (i.p.) injected into C57BL6/J mice (RRID:IMSR_JAX:000664). Tumour establishment was confirmed using an IVIS Lumina X5 imaging system, with luciferin bioluminescence images acquired and analysed by in vivo imaging software. Following tumour establishment, mice were treated with CHK1i (SRA737) + LDHU as described previously [31]. Briefly, mice were treated every other day for 3 days per week for 3 weeks from day 21 after tumour implantation with vehicle (10% DMSO, 5% Tween80, 5% PEG400 in clinical-grade saline) or 50 mg/kg CHK1i (SRA737, Sierra Oncology, San Mateo, CA, USA) combined with 100 mg/kg HU in the vehicle by oral gavage, then 4 h later by i.p. injection of 50 mg/kg HU (in saline). To examine the influence of CD8^+^ T cells on treatment responses, anti-CD8β (Lyt 3.2, BioXcell) or appropriate isotype control injections were initiated 3 days prior to CHK1i+LDHU treatments and then repeated weekly. At the experimental endpoint, ascites fluid was collected, and tumours in omentum and other organ sites were dissected in a double-blinded manner.

### Immune Profiling

Tumours in omentum were harvested, minced and treated with DNASe1 and collagenase IV for 30 min, then pushed through a 40 μm cell strainer to generate a single-cell suspension. Cells were blocked using Fc block then stained with Live/dead Aqua (Thermo Fisher Scientific, Waltham, MA, USA) and a panel of conjugated antibodies in 1:100 dilution for immune cell profiling (CD45.2-PE dazzle, CD3-FITC, TCRβ-PE, CD8α-BV605, CD4-BUV395, NK1.1-PE-Cy7, CD11b-BV421, F4/80-BV711, Gr-1-PercpCy5.5, Ly6G-AF700, MHCII-APC-Cy7, CD19-BV785) (Supplementary Table S3). FoxP3 (FoxP3-AF647) was detected using the FoxP3 Staining Kit (eBioscience) as per the manufacturer’s instructions. In some experiments, the lymphoid and myeloid markers were separated, PD-L1-BV711 and CD86-APC added to the myeloid panel and CD25-BV421 added to the lymphoid panel. Flow-Count Fluorospheres (Beckman Coulter, Miami, FL, USA) were used for total cell counts. Stained cells were analysed using an CYTEK Aurora Spectral Flow Cytometer (Cytek Biosciences) with SpectroFlo software, RRID:SCR_025494. Spectral unmixing was performed as described in the Supplementary Material.

### Statistical analysis

All statistical analyses were performed using GraphPad Prism 9 (RRID:SCR_002798). Bar graphs display mean values and standard deviation (SD).

### Ethics approval and consent to participate

Human ethics was provided by the Mater Misericordiae Ltd Human Research Ethics Committee (MML HREC) for Project Id: 29596, approval PRGRPT/MML/29596. Informed consent was obtained from each patient for their ascites samples, and the study was performed in accordance with the Declaration of Helsinki. Animal ethics was obtained from The University of Queensland Molecular Biosciences Animal Ethics Committee – 2021/AE000249.

## Results

**Fig. 1:**
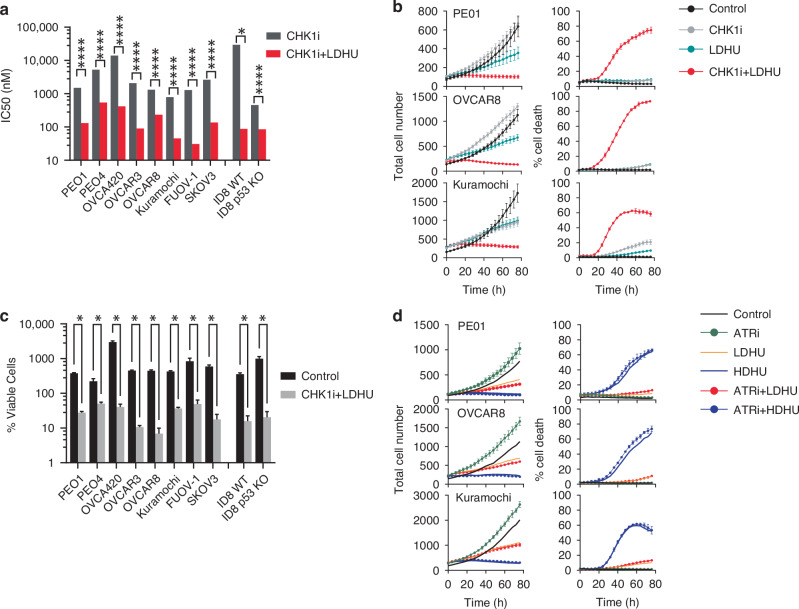
Ovarian cancer cell lines are sensitised to CHK1i combination. **a** IC50 values of the indicated cell lines for CHK1i (SRA737) with and without 0.2 mM HU (HU). The data are from 4 technical and 2 biological replicates. *p* values from extra sum of squares test of best fit. * *p* > 0.05, ****p > 0.0001. **b**–**d** The indicated HGSOC cell lines (see Tables S1), were treated under the following conditions: Control (vehicle), CHK1i (1 µM SRA737), LDHU (low-dose HU, 0.2 mM), HDHU (high-dose HU, 2 mM), ATRi (2 µM VE-821), CHK1i+LDHU, ATRi+LDHU, and ATRi+HDHU. Cells were monitored using the IncuCyte system for up to five days. Total cell numbers and % cell death were calculated as outlined in the Methods. % viable cells was calculated from number of viable (Sytox negative) cells in final time point of the Incucyte experiments/cell number in the initial time point. Data represent the mean ± SD of 6–30 replicates from 3 independent experiments. * *p* < 0.0001 using two way ANOVA with Sidak’s multiple comparison test.

**Fig. 2:**
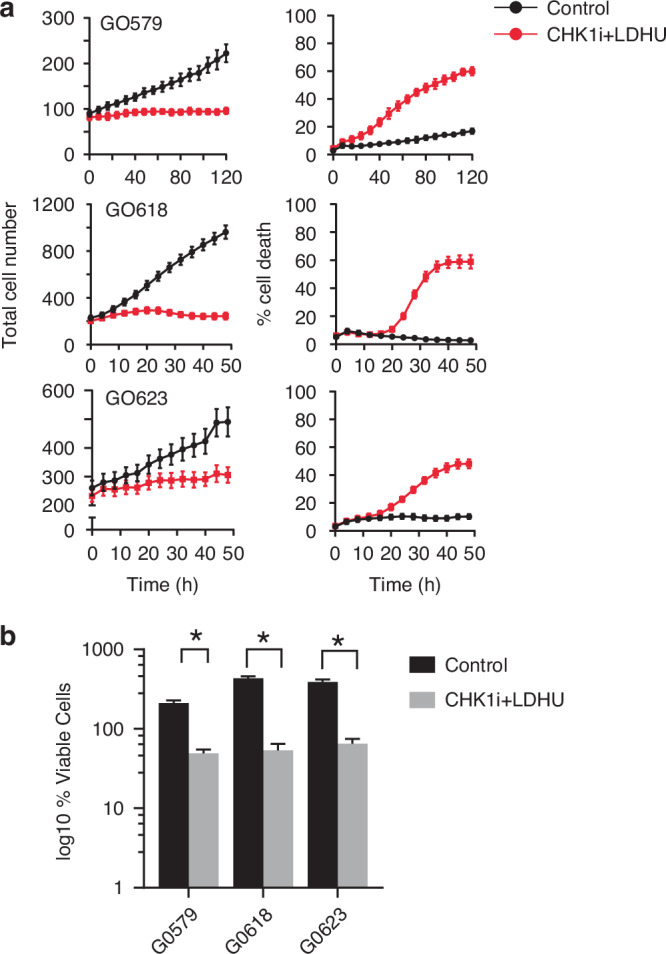
Primary ovarian cancer cells are sensitised to CHK1i combination. **a** The indicated HGSOC primary cells derived from patient ascites samples (see Tables S2), were treated with Control (vehicle) or CHK1i (1 µM SRA737) plus LDHU (low-dose HU, 0.2 mM), Cells were monitored using the IncuCyte system for up to five days. **b** % viable cells was calculated from number of viable (Sytox negative) cells in final time point of the Incucyte experiments/cell number in the initial time point. Data represent the mean ± SD of 6–30 replicates from 3 independent experiments. * *p *< 0.001 using two way ANOVA with Sidak’s multiple comparison test.

We assessed the efficacy of the CHK1i+LDHU combination across a panel of established mouse and human ovarian cancer cell lines and in tumour cells recently derived from HGSOC patient ascites, including two who recurred after chemotherapy and thus clinically chemo-resistant. The panel is representative of chemo-sensitivity, *BRCA* mutation and homologous recombination deficiency (HRD) status found in HGSOC patients (details in Table S1 and S2). Dose-response assays using the clinically tested CHK1i (SRA737) demonstrated that combination with 0.2 mM HU reduced IC50 for CHK1i 5-25 fold in all tested cell lines, with IC_50_ values below 1 μM for the CHK1i (Fig. 1a). This is significantly lower than the reported 6 μM Cmax in patients [30]. Based on these findings, a combination of 1 μM CHK1i and 0.2 mM HU was selected. The synergy of this combination was assessed in three cell lines. As single agents, CHK1i and LDHU treatment had little or modest effects on cell proliferation and cell killing, whereas the combination blocked proliferation and promoted high levels of cell killing in the tested cancer cell lines (Fig. 1b). This clearly demonstrated the individual components had little anti-proliferative effect, and the synergy of the combination. The degree of cell killing achieved by CHK1i+LDHU was similar to CHK1i combination with 2 mM HU (HDHU) (Supplementary Figure S2). Cell killing was calculated as percentage of dead cells of the total cell number in each frame and underrepresents level of the cell killing relative to the either starting cell number or control cell numbers at the same time point. By calculating the percentage of viable cells in the final frame in the control and treated samples relative to the first frame of the time lapse sequence, treated samples had final cell viability >40% of the initial cell number across a larger panel of human and murine OvCa lines (Fig. 1c and Supplementary Figure S3). The sensitivity did not appear to be determined by the genotype or chemo-sensitivity as the most sensitive cell lines (those with lowest viable cell count with treatment) OVCAR3 and OVCAR8 were both chemo-resistant, and BRCA deleted and wild type, respectively (Table S1). LDHU alone produced a modest reduction in proliferation (Fig. 1b), differentiating it from the ATR-dependent checkpoint response triggered by agents such as HDHU [37]. ATR inhibitor had no effect when combined with LDHU and had modest effect on cell killing when combined with HDHU (Fig. 1d), similar to that observed with CHK1i+HDHU (Supplementary Figure S2). Tumour cells cultured from patient ascites samples (GO579, GO618, GO623) were also sensitive to the combination (Fig. 2), demonstrating similar sensitivity to CHK1i+LDHU in recent patient derived cells. Together, these data demonstrate that CHK1i+LDHU effectively induces cell death across a range of ovarian cancer models, irrespective of genetic background, HRD status, or chemotherapy resistance.

**Fig. 3:**
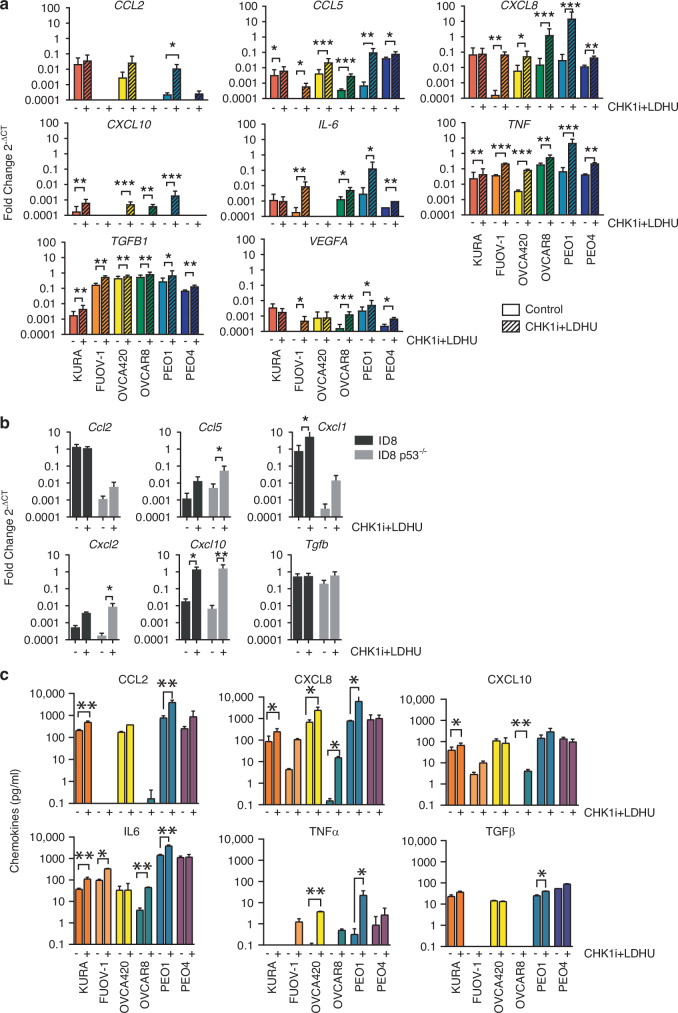
CHK1i combination treatment induces proinflammatory responses in ovarian cancer cells. The indicated ovarian cancer cell lines were treated with (+) or without (−) 1 µM CHK1i (SRA737) + 0.2 mM HU for: **a**, **b** 24 h, followed by cell harvesting and measurement of the mRNA for indicated cytokines/chemokines using qRT-PCR, and (**c**) 48 h, followed by harvesting supernatants for cytokine level measurement. The levels of the qRT-PCR products are shown relative to control. The qRT-PCRF data are representative of 2–4 independent experiments with at least three replicates. Chemokine levels were measured in 2 individual experiments with 2 replicates. Absence of values indicates no signal detected. *P* values calculated by two way ANOVA using Uncorrected Fisher’s Least Significant Difference (LSD) test for each chemokine. * *p* < 0.05, ** *p* < 0.01, *** *p* < 0.001.

CHK1i+LDHU induced replication stress in ovarian cancer cells PE01, OVCAR8, Kuramochi, FUOV-1 and G0618, as evidenced by the slower migrating RPA2 band that is indicative of RPA2S4/8 phosphorylation [38], and increased DNA damage, indicated by elevated γH2AX levels. OVCA420 have elevated markers without treatment suggesting high levels of endogenous replication stress (Supplementary Figure S4), consistent with previous findings [10, 11]. DNA damage is known to increase pro-inflammatory cytokine and chemokine expression [39]. CHK1i+LDHU treatment significantly increased the expression of pro-inflammatory signals *CCL5*, *CXCL8*, *CXCL10, IL-6*, and *TNF* at RNA levels across most of the human ovarian cell lines, although there was considerable variability in the responses with FUOV-1 and PEO4 not expressing detectable *CXCL10* and OVCA420 not expressing detectable *IL-6* (Fig. 3a). *CCL2* was also upregulated with treatment in OVCA420 and PEO1 cells lines. In murine ovarian cancer lines, *Ccl5*, *Cxcl10*, and murine equivalents of *CXCL8* (*Cxcl1* and *Cxcl2*) were similarly upregulated with treatment, with the trends in changes of expression being similar although absolute levels differed for *Ccl2* and *Cxcl1* (Fig. 3b). Expression of anti-inflammatory *TGFb* and *VEGFA* were also modestly increased with treatment in the human cell lines, but not in the murine lines (Fig. 3a, b). The changes in levels of the secreted proteins (Fig. 3c) generally mirrored the RNA expression patterns (Fig. 3a), with pro-inflammatory signals (CCL2, CXCL8, CXCL10, IL-6 and TNFα) increasing with treatment, while levels of free active TGF-β1, the biologically active form of the cytokine, remained unchanged. Although the levels of the secreted murine cytokines/chemokines were not assessed, it was assumed they mirrored what was observed in human cell lines. Overall, CHK1i+LDHU promoted a pro-inflammatory microenvironment, upregulating expression of CCL2, CCL5, CXCL10 and TNFα while leaving the anti-inflammatory cytokine TGFβ largely unaffected.

**Fig. 4:**
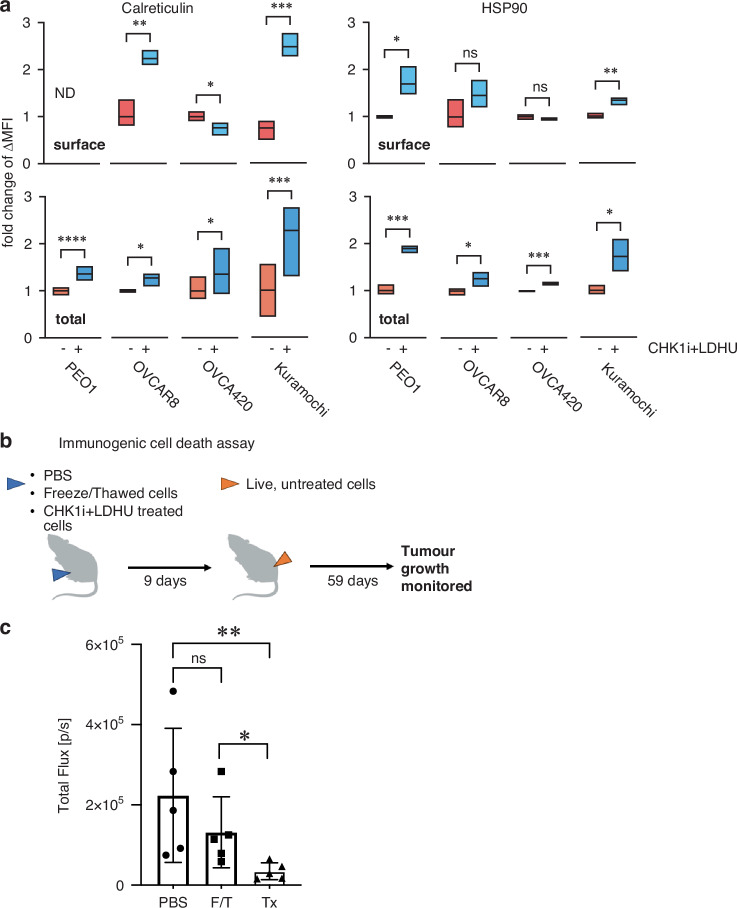
CHK1i combination treatment promotes ICD in ovarian cancer models. Human ovarian cancer cell lines were treated with or without 1 μM CHK1i (SRA737) + 0.2 mM HU and harvested at 24 h for analysis of calreticulin and HSP90 on live cells. **a** Fold change in cell surface (top) or whole cell total (bottom) expression levels of calreticulin and HSP90. Data are representative of three independent experiments. ND, none detected. **b**, **c** C57BL/6 J mice were immunised with ID8-p53^−/−^ cells treated in vitro 1 µM CHK1i (SRA737) + 0.2 mM HU (Tx) for 24 h. Freeze-thaw killed (F/T) ID8-p53^−/−^ cells were used as a negative control for ICD. At 9 days after immunisation mice were rechallenged with live ID8-p53^−/−^ cells into the opposite flank and tumour burden was monitored by IVIS in vivo. *N* = 5 mice each treatment. Statistical analysis using unpaired *t*-test with Welch’s correction * *p* < 0.05, ** < 0.01, *** < 0.001, **** < 0.0001.

In addition to its direct cytotoxicity and induction of pro-inflammatory signals, we investigated whether CHK1i+LDHU treatment could further modulate the TIME by triggering immunogenic cell death (ICD). The cell surface expression of damage-associated molecular patterns (DAMPs) calreticulin and HSP90 were assessed in a representative panel of cell lines. There was significantly increased surface expression of calreticulin in OVCAR8 and Kuramochi, and increased surface HSP90 in PEO1 and Kuramochi, although increased total (intracellular and surface) expression of both proteins was observed in all cell lines with treatment (Fig. 4a). To determine the immunogenic potential of CHK1i+LDHU in vivo, luciferase-labelled mouse ID8-p53^−/−^ cells treated in vitro with CHK1i+LDHU for 24 h were used to inoculate immunocompetent mice. Freeze-thawed tumour cells were used as a negative control for ICD response. After 9 days, all mice were challenged with live, untreated ID8-p53^−/−^ tumour cells injected into the opposite flank and tumour growth was monitored (Fig. 4b). Tumour burden, assessed by bioluminescence imaging, was similar in the PBS control and freeze-thawed cell inoculated mice, whereas mice inoculated with CHK1i+LDHU-treated ID8-p53^−/−^ cells were significantly protected (Fig. 4c).

**Fig. 5:**
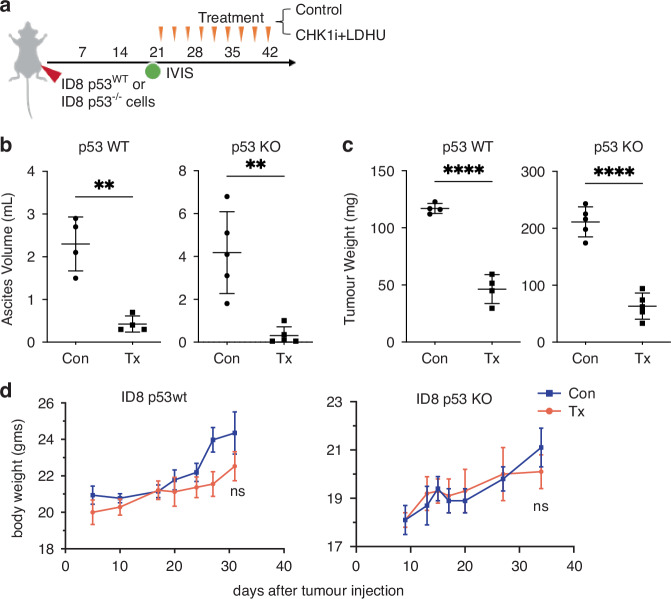
CHK1i combination treatment effectively controls ascites accumulation and tumour growth of ID8-p53^WT^ and ID8-p53^−/−^ models. **a** Syngeneic mouse ovarian cancer models ID8-p53^WT^ and ID8-p53^−/−^ were established in immunocompetent mice (*n* = 5 per treatment group). Once tumour engraftment and dissemination throughout the abdominal cavity were confirmed by IVIS imaging, mice were treated with vehicle control or CHK1i combination therapy, as previously described [31]. **b** Volume of ascites, and (**c**) tumour burden at endpoint in mice treated (Tx) or untreated (Con) with three weekly cycles of CHK1i combination therapy. **d** Body weight measurements following tumour cell injection over the course of the study. Statistical analysis using unpaired two tailed *t*-test for each model ** *p* < 0.01, **** < 0.0001.

**Fig. 6:**
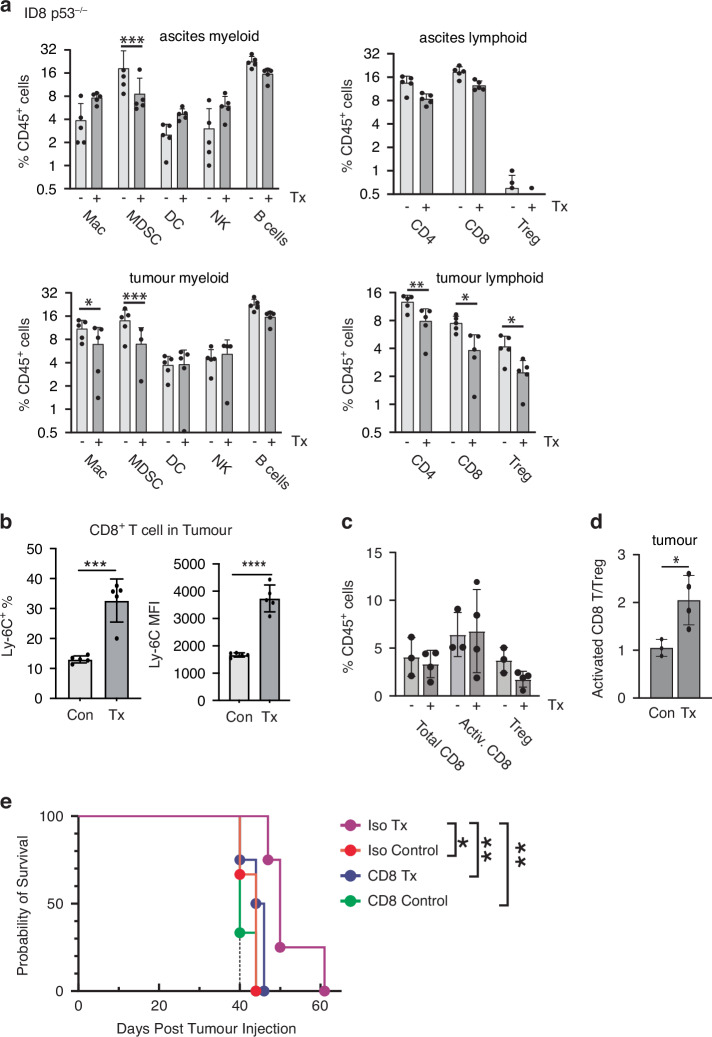
CHK1i combination induced anti-tumour immune response is dependent on CD8^+^ T cells. ID8 p53^−/−^ tumours were established in immunocompetent mice with treatment commenced day 21. **a** Ascites and omental tumour samples from Fig. 5 were processed and stained with immune cell markers. The relative abundance of the indicated major immune cell types in either the myeloid (MDSCs, macrophages, DCs, NK cells, B cells), or lymphoid cell compartments (CD4^+^ and CD8^+^ T cells, Tregs) were assessed using the gating strategy in Supplementary Figure S5A. Statistical analysis using two way ANOVA and Uncorrected Fisher’s LSD. **b** Gr-1expression on CD8^+^ T cells, percent Gr-1^+^ cells gated from CD8^+^ T cells and mean fluorescence intensity (MFI) of Gr-1 in ID8-p53^−/−^ tumours. **c** The levels of total CD8^+^ and activated CD8^+^ (Ki67^+^, PD-1^+^) and Treg cells from a second ID8 p53^−/−^ experiments similar to **a** (gating strategy Supplementary Figure S5B), (**d**) the ratio of activated CD8^+^ to Treg cells is shown. **e** In ID8-p53^−/−^ tumour bearing mice, antibody injections, either isotype control or anti-CD8β were started with CHK1i+LDHU treatment (day 21) and then repeated on day 28 and day 35 alongside l CHK1i+LDHU treatment. Kaplan-Meier graph showing the survival of mice treated with: Iso = Isotype mAb, CD8 = anti-CD8 mAb, Control = Placebo control, Tx = CHK1i+LDHU. Statistical analysis using two tailed unpaired *t*-test * *p* < 0.05, ** < 0.01, *** < 0.001, **** < 0.0001. For survival, used log rank test.

Based on these finding, we further investigated the in vivo therapeutic efficacy of CHK1i+LDHU. Two different murine ovarian cancer models, luciferase-labelled ID8-p53^WT^ and ID8-p53^−/−^ tumours were assessed using syngeneic C57BL/6 J mice. Low dose hydroxyurea had only modest effects on tumour cell growth but no toxicity in vitro (Fig. 1b), has been reported to have no effect on tumour growth [38], and higher doses had no effect on tumour growth in ovarian cancer patients [40]. CHK1i (SRA737) was reported to effect ovarian cancers as a single agent [30], but had no effect in vivo as a single agent at the dose used here (Supplementary Figure S5). Three weeks of treatment with 3 days/week CHK1i+LDHU (Fig. 5a) effectively blocked ascites accumulation and tumour growth in both models (Fig. 5b, c) but had little effect on mouse body weights (Fig. 5d). The slightly lower body weights of the treated mice accounted for the reduced ascites and tumour weight. To assess whether the pro-inflammatory cytokine and chemokine expression triggered by the treatment altered the TIME, immune profiling was performed using a panel of flow cytometry markers for myeloid and lymphoid cell types. In ID8-p53^−/−^ tumours, immunosuppressive MDSCs and Tregs, major drivers of immune suppression in HGSOC TIME [9], were significantly decreased (Fig. 6a and Supplementary Figure S6A for gating strategy) which was also found in ID8 p53^WT^ tumours (Supplementary Figure S7A). The decreased MDSC in the tumour mirrored changes in the ascites. Few Tregs were found in ascites. There were only minor changes in the abundance of CD8^+^ T cells with CHK1i+LDHU treatment in either model. The reduction in the CD4^+^ T cells abundance reflected the reduction in the Treg levels (Fig. 6a and Supplementary Figure S7A). Ly-6C expressing CD8^+^ T cells (detected by the Gr-1 antibody and more cytotoxic than normal effector T cells [41]) were significantly increased with treatment, and Ly-6C expression was 3-fold higher in this subset compared to non-treated tumours (Fig. 6b). Using a different panel of T cell markers in a second ID8-p53^−/−^ experiment, the total and activated CD8^+^ T cell level (CD8^+^, Ki67^+^, PD-1^+^; Supplementary Figure S6B for gating strategy), again little change in CD8^+^ T cells levels were noted, and no significant increase in activated T cells using these markers, but the ratio of activated CD8^+^ T cells and Tregs improved significantly (Fig. 6c, d).

To determine whether cytotoxic CD8^+^ T cells are involved in the CHK1i combination induced anti-tumour responses, CD8^+^ T cells were specifically deplete in the ID8-p53^−/−^ model using anti-mouse CD8β (Lyt 3.2, Clone 53–5.8). The 53–5.8 antibody has been shown to achieve complete depletion of CD8^+^ T cells in vivo without affecting CD8^+^ CD11c^+^ dendritic cells which primarily express the CD8αα homodimer [42]. Depletion of CD8^+^ T cells using the anti-mouse CD8β antibody (Supplementary Figure S7B) abolished the ability of CHK1i+LDHU to control the tumour growth, demonstrating a key role for CD8^+^ T cells in the combination’s mechanism of action (Fig. 6e).

## Discussion

Current chemotherapy for HGSOC is initially effective but will inevitably lead to resistance and comes with very significant toxicity to the patients [43]. Here we demonstrate that a synergistic combination of subclinical doses of CHK1i and low dose HU is effective in killing a high proportion of HGSOC cell lines and new patient derived models in vitro, irrespective of genotype, HRD status or chemo-sensitivity. The broad effectiveness of the CHK1i+LDHU combination has also been observed in melanoma and NSCLC models [10]. A combination of CHK1i with low dose gemcitabine has also been shown to be effective in SCLC where it also triggers a CD8^+^ T cell dependent immune response [44]. However, even low doses of gemcitabine can trigger the ATR/CHK1-dependent S phase checkpoint arrest rather than the unique ATR-independent, CHK1-dependent replication origin reorganisation that is triggered by LDHU [10, 26]. Notably, unlike the higher concentrations of HU or gemcitabine that are effective as single agents and trigger an S phase arrest, the low dose HU used here triggers a CHK1i-dependent response without causing growth arrest either in vitro or in vivo [10, 27, 29]. Of note, normal tissue, especially the proliferating cell in the colonic crypts and immune cells that are responsible for the many toxic side effects of chemotherapy are not adversely affected by CHK1i+LDHU in vitro or in vivo [10, 31]. The broad effectiveness of CHK1i+LDHU is likely to be due to replication stress inherent in the development of HGSOC [14].

The synergistic CHK1i+LDHU combination can promote a pro-inflammatory environment that is the result of both pro-inflammatory cytokine/chemokine expression and DAMPs released as a consequence of tumour cell ICD [45]. Targeted therapies such as PARP inhibitors can also trigger ICD and pro-inflammatory responses [45, 46], but are associated with high rates of high-grade haematological toxicity including reduction in lymphocytes [47], suggesting that the immunomodulation by PARPi is negated by a sub-optimal immune response. This may account for the lack of efficacy of combination of PARPi with immunotherapy [47]. The relative lack of deleterious effect of CHK1i+LDHU on either T cell proliferation in vitro or on a T cell dependent immune response in vivo [31] provides strong evidence that this combination has minimal negative impact on the immune function, making an anti-tumour immune response a viable outcome.

Overcoming the immunosuppressive state common in ovarian cancer requires an anti-tumour treatment capable of targeting multiple components. Beyond its direct tumour cell–killing effects, CHK1i+LDHU induced expression of pro-inflammatory chemokines and cytokines such as CCL2, CCL5, CXCL10 and TNF-α. Although each cell line increased expression of different combinations of cytokines/chemokines with treatment, the combination effect was an overall shift towards a pro-inflammatory environment may have played a pivotal role in modulating immune cell recruitment and activity. CXCL10 has been shown to recruit and restimulate T cells, including effector CD8^+^ T cells [48]. Similarly, CCL2 and CCL5 have been implicated in the recruitment of antigen-presenting cells including dendritic cells (DCs) to inflammatory sites, further supporting their role in enhancing antigen presentation and T cell activation [49]. High CCL2 levels promote a pro-inflammatory Th1/Th17 phenotype, while CCR2 deficiency shifts the immune balance toward Tregs [50]. High CCL5 expression promotes the recruitment of CD8^+^ T cells, NK cells, and activated macrophages [51], and CCL5 has been shown to recruit CCR5^+^ dendritic cells to enhance CD8^+^ T cell priming and sustain anti-tumour responses [52]. TGF-β can promote immunosuppression by converting CD4^+^ T cells to Tregs [53]. Although *TGFB1* was abundantly expressed in the tested cell lines and modestly upregulated by CHK1i+LDHU treatment, only low levels of free active TGF-β1 were detected. This suggests that increased expression of the latent form could result in increased active TGF-β1 in the presence of maturing proteases [54].

CHK1i+LDHU significantly reprogrammed the TIME by reducing immunosuppressive MDSCs and Tregs - critical barriers to effective immune surveillance in HGSOC [55], and by activation of CD8^+^ T cells. We demonstrated that much of the treatment induced anti-tumour response was dependent on CD8^+^ T cells. The marked loss of tumour control following CD8^+^ T cell depletion was surprising given the combination’s efficacy in killing tumour cells in vitro. Similarly, treatment response was lost in syngeneic melanomas grown RAG1^−/−^ mice which lack adaptive immunity [31], despite being highly effective in immunocompromised Nude mice [10]. These findings suggest that the anti-tumour effect induced by CHK1i+LDHU is not solely due to direct tumour cell killing but also relies on CD8⁺ T cells, providing strong evidence that this treatment harnesses the immune system to combat the tumour – an effect that is particularly important in HGSOC, where most patients do not respond to immunotherapy.

In this study, we demonstrated that CHK1i+LDHU induces a robust anti-tumour immune response, modulating both innate and adaptive immune responses. Current immunotherapies used as single agents are effective in tumours that have already triggered an immune response, the so called “hot” tumours, but are ineffective in “cold” tumours where no prior immune response exists [56]. The ability of CHK1i+LDHU to induce ICD and enhance expression of pro-inflammatory cytokines/chemokines reshapes the TIME, reducing immunosuppressive elements such as MDSCs and Tregs, and importantly, establishes a potent CD8^+^ T cell-mediated anti-tumour response regardless of its initial status. A limitation of this study is that all in vivo experiments were performed in single mouse strain, C57-BL/6 J, which essentially mimics a single patient in terms of the genetic diversity of the immune response. Furthermore, this model is BRCA1/2 wildtype and thus only reflects the homologous recombination competent HGSOC patient population. Future studies will expand into humanised mouse models using homologous recombination components and defective patient derive xenograft models. Of the cell lines in this study, those with BRCA mutations were limited to the BRCA2 gene, so investigation of treatment sensitivity in other homologous recombination defective models would be of interest.

In conclusion, synergistic CHK1i+LDHU combination therapy represents a promising strategy for treating HGSOC by directly killing tumour cells and importantly, reprogramming the immune microenvironment to promote an effective anti-tumour immune response in vivo. By including primary tumour cells derived from patient ascites alongside a diverse panel of established human ovarian cancer cell lines, this study provides strong evidence that the efficacy of CHK1i+LDHU extends to clinically relevant, heterogeneous tumour contexts. The high sensitivity in diverse HGSOC models, even in models derived from chemo-resistant patients suggests that the CHK1i combination could be an effective option for patients who have exhausted standard treatment options.

## Supplementary information

Supplementary Information

## Data Availability

The original contributions presented in this study are included in the article/supplementary material. Further inquiries can be directed to the corresponding author(s).
