# Supplementary material for: Using targeted therapy to promote a pro-inflammatory tumour microenvironment and anti-tumour immune response in high grade serous ovarian cancer
Source: Br J Cancer. 2026 Apr 7;134(12):1830–40. doi: 10.1038/s41416-026-03416-y (PMC13226670; doi:10.1038/s41416-026-03416-y)

Supplementary Information

Supplementary Methods

HRD functional assay

The cell lines were seeded on sterilized Poly-l-lysine treated coverslips in 6-well plates and left to settle for 24-48hrs. Cells were irradiated with 10 Gy and after 4hrs, were fixed with 4% paraformaldehyde (Sigma Aldrich) for 20 min and permeabilized with 0.1 % Triton X-100 for 15 min. Coverslips were blocked in 3% BSA + 0.1% saponin (Sigma, S54521-25g) at RT for 1 hour. The primary antibody incubation with anti-RAD51 antibody and anti-γH2AX antibody (Table Supplementary Table 3) in blocking buffer overnight at 4°C. Coverslips were washed with x1 PBS before the secondary antibodies (Table Supplementary Table 3) incubated for 1h at RT. Coverslips were mounted on a cleaned glass slide using Prolong Diamond Antifade Mountant (Invitrogen, P36961). Images were captured initially with an upright motorized microscope (Olympus BX63 upright epifluorescence) using 20x and then image with the laser scanning confocal microscope (Olympus FV3000) at 40x magnification. Images were visualized and analyzed using ImageJ software and figures were created using Adobe Illustrator.

Patient ascites-derived tumour cells

HGSOC patient ascites were collected from patients undergoing palliative care from Mater Hospital Brisbane. Patients provided informed consent for sample collection and use under ethical approval provided by Mater Misericordiae Ltd Human Research Ethics Committee (MML HREC) project #29596. Tumour cells were isolated from ovarian cancer patient ascites (as described in Supplementary Table S2) through centrifugation at 340 g for 10 minutes, followed by red blood cell lysis with ACK buffer (10 mM Tris-Cl, 5 mM $MgCl_2$, 10 mM NaCl). The isolated cells were resuspended and cultured for 24 hours in complete DMEM media. This

initial culture allowed most of the fibroblast population to adhere. Subsequently, all media was transferred into a new flask and cultured for an additional 48 hours to allow tumour cell adherence. The media was then changed to a supplemented mixture consisting of 33% Medium 199 (Gibco), 33% DMEM/F12 (Gibco), 33% RPMI (Gibco) with 10% heat-treated fetal bovine serum (FBS; Bovogen), 1% Insulin-Transferrin-Selenium (Gibco), 25 ng/ml Cholera Toxin (Sigma-Aldrich), 0.5 µg/ml Hydrocortisone (Sigma-Aldrich), 10 ng/ml Epidermal Growth Factor (Sigma-Aldrich), 10 ng/ml Fibroblast Growth Factor (Sigma-Aldrich), and antibiotic-antimycotic. After reaching confluency, differential trypsinization was performed to remove any remaining fibroblasts by adding 0.25% Trypsin-EDTA (Gibco) for 40 seconds and removing the supernatant containing the fibroblasts. Cell cultures were maintained in a Binder low oxygen incubator at 37°C, 5% CO_2, and 2% O_2.

Spectral unmixing for Cytek flow cytometry

Spectral unmixing was performed in SpectroFlo (RRID:SCR_025494) using the manufacturer's standard unmixing workflow. Single-stained compensation controls for every fluorochrome in the panel were used to generate reference emission spectra. An unstained cell control was included to capture and model the sample-specific autofluorescence profile, which was incorporated automatically by SpectroFlo's autofluorescence extraction algorithm during unmixing. This approach allowed subtraction of the autofluorescence signal across the full spectrum, improving resolution of dim markers and ensuring accurate separation of fluorochromes. All samples were unmixed using the same reference controls within the same acquisition session to maintain consistency and reproducibility. Acquired data were analysed using FlowJo software, RRID:SCR_008520.

Tables

Supplementary Table S1: Characteristic of the ovarian cancer cell lines used.

Cell line	Disease	Tissue Derived from	Treatment history	BRCA1/2 status	HRD Status	Additional mutations
**FUOV-1**	High grade ovarian serous adenocarcinoma	Primary tumour	Chemo naïve[1]	WT	Negative	TP53 mutated
**Kuramochi**	High grade ovarian serous adenocarcinoma	Ascites	N/A	BRCA2 mut	Positive	TP53 mutated
**OVCAR8**	High grade ovarian serous adenocarcinoma	Primary tumour	Progression on Carboplatin	WT	Negative	TP53, KRAS, ERBB2, CTNNB1
**OVCAR3**	High grade ovarian serous adenocarcinoma	Ascites	The patient previously received cyclophosphamide, cisplatin, and doxorubicin treatment[2]	BRCA2 deleted	Negative	TP53 mutated
**OVCA420**	Ovarian serous adenocarcinoma	Primary tumour	N/A	WT	Negative	TP53 mutated
**PEO1**	Ovarian cystadenocarcinoma	Ascites	The patient previously received cisplatin, 5-fluoruoracil and chlorambucil treatment and developed clinical resistance to chemotherapy.[3]	BRCA2 mut	Positive	TP53 mutated
**PEO4**	Ovarian cystadenocarcinoma	Ascites	Another cell line derived from the same patient as PEO1 [3]	BRCA2 revertant	Negative	TP53 mutated
**SKOV-3**	Ovarian serous cystadenocarcinoma	Ascites	N/A	WT	Negative	TP53 deleted
**ID8 p53 WT**		Mouse primary tumour		WT	Negative	None
**ID8 p53 KO**		Mouse primary tumour		WT	Negative	TP53 deleted

This information was drawn Cellosaurus (RRID:SCR_013869) and Cancer Cell Line Encyclopedia databases. In addition, we performed a functional HRD analysis using RAD51 focus formation after ionising radiation (See Supplementary Methods).

Supplementary Table S2:

Patient number	Disease	Treatment history	BRCA1/2 status	HRD status genetics	Additional mutations
GO579	HGSOC	Carboplatin and Paclitaxel	No pathogenic	Positive	TP53
GO618	HGSOC	Pre-treatment	No pathogenic	Negative	TP53
GO623	HGSOC	Carboplatin and Paclitaxel	No pathogenic	Negative	None reported

Patients GO579 and GO623 were palliative care patients after disease relapse, GO618 was from a patient prior to neo-adjuvant Carboplatin and Paclitaxel treatment. Genetic testing (whole exome) for *BRCA1* and *BRCA2* mutations and copy number, and HRD genetic testing including *PALB2, RAD51C, RAD51D, TP53* revealed no pathogenic mutations. In addition, we have performed HRD testing using RAD51 foci formation after ionising radiation exposure to functionally assess the HRD status of the cell lines derived from these patient's ascites.

Supplementary Table S3: Summary of antibodies used

Antibody	Supplier (Cat#)	RRID
Immunoblots		
RPA2	Cell Signalling Technology (52448)	AB_2750889
γH2AX	Cell Signalling Technology (5438)	AB_10707494
Alpha-Tubulin	Rockland (600-401-880)	AB_2137000
Immunofluorescence		
RAD51	Cell Signalling Technology (65653)	AB_3718052
γH2AX	Merck (05-636)	AB_309864
AF555 goat anti-mouse IgG	Invitrogen (A21424)	AB_141780
Cy5 goat anti-rabbit IgG	Invitrogen (A10523)	AB_2534032
Flow Cytometry		
ICD Panel		
HSP90β-PE	Enzo Life Sciences (ADI-SPA-844PE-050)	AB_11180662
Calreticulin-Alexa Fluor 645	Abcam (ab196159)	AB_2819061
Immune profiling panel		
TruStain FcX™ (anti-mouse CD16/32)	Biolegend (101320)	AB_1574975
CD11b- PercpCy5.5	Biolegend (101228)	AB_893232
CD11b-BV421	Biolegend (101236)	AB_11203704
CD11b-BV650	Biolegend (101259)	AB_2566568
CD11c-BV785	Biolegend (117336)	AB_2565268
CD11c-PacificBlue	Biolegend (117322)	AB_755988
CD19-BV605	Biolegend (115540)	AB_2563067
CD19-BV785	Biolegend (115543)	AB_11218994
CD206-PE	Biolegend (141705)	AB_10896421
CD25-BV421	Biolegend (102033)	AB_10895908
CD3-FITC	Biolegend (100204)	AB_312661
CD4-BUV395	Invitrogen (363-0042-82)	AB_2920941
CD4-Alexa Fluor 700	Biolegend (100536)	AB_493701
CD45.2-PE/Dazzle 594	Biolegend (109846)	AB_2564177
CD45.2-BV650	Biolegend (109836)	AB_2563065
CD45-PE-CF594	BD Biosciences (562452)	AB_11152958
CD8α-BV605	Biolegend (100744)	AB_2562609
CD8A-BV605	Biolegend (100744)	AB_2562609
CD8b-APC-Cy7	Biolegend (126620)	AB_2563951
CD86-APC	Biolegend (105012)	AB_493342
F4/80-BV711	Biolegend (123147)	AB_2564588
F4/80-PeCy7	Biolegend (123114)	AB_893478
FOXP3-Alexa Fluor 647	Biolegend (126408)	AB_1089115
Gr-1-PercpCy5.5	Biolegend (108427)	AB_893561
Ki67-PE-Cy7	Biolegend (151217)	AB_2910305
Ly6C-APC	Biolegend (128015)	AB_1732087
Ly6C-APCe780	eBiosciences (47-5932-80)	AB_2573991
Ly6G-Alexa Fluor 700	Biolegend (127622)	AB_10643269
MHCII-APC-Cy7	Biolegend (107628)	AB_2069377

MHCII-PacificBlue	Biolegend (107620)	AB_493527
NK1.1-PE-Cy7	Biolegend (108714)	AB_389364
NK1.1-PE-Cy7	eBiosciences (25-5941-82)	AB_469665
PD-L1-BV711	Biolegend (124319)	AB_2563619
PD-L1-PE	Biolegend (155404)	AB_2728223
TCRβ-PE	Biolegend (109207)	AB_313430
TCRβ-FITC	Biolegend (109206)	AB_313429
TCRβ-Percp-Cy5.5	Biolegend (109228)	AB_1575173

Supplementary Table S4: RT-qPCR primers

Primer (Mouse)	Sequence (5'-3')	Primer (Human)	Sequence (5'-3')
**FM1 ACTIN**	CGAATCATGAGCATTGTAGAC	**FM1 CCL2**	AGACTAACCCAGAAACATCC
**BM1 ACTIN**	GTAATTCTTATCTCCAGCCAG	**BM1 CCL2**	ATTGATTGCATCTGGCTG
**FM1 CCL2**	CAAGATGATCCCAATGAGTAG	**FM1 CCL5**	ACTTGCCTCCCCATATTC
**BM1 CCL2**	TTGGTGACAAAAACTACAGC	**BM1 CCL5**	AAGAGTTGATGTACTCCCG
**FM1 CCL5**	AGGAGTATTTCTACACCAGC	**FM1 CXCL8**	GTTTTTGAAGAGGGCTGAG
**BM1 CCL5**	CAGGGTCAGAATCAAGAAAC	**BM1 CXCL8**	TTTGCTTGAAGTTTCACTGG
**FM1 CXCL1**	AAAGATGCTAAAAGGTGTCC	**FM1 CXCL10**	AAAGCAGTTAGCAAGGAAAG
**BM1 CXCL1**	GTATAGTGTTGTCAGAAGCC	**BM1 CXCL10**	TCATTGGTCACCTTTTAGTG
**FM1 CXCL2**	GGGTTGACTTCAAGAACATC	**FM1 IL-6**	GCAGAAAAAGGCAAAGAATC
**BM1 CXCL2**	CCTTGCCTTTGTTCAGTATC	**BM1 IL-6**	CTACATTTGCCGAAGAGC
**FM1 CXCL10**	AAAAAGGTCTAAAAGGGCTC	**FM1 TNF**	AGGCAGTCAGATCATCTTC
**BM1 CXCL10**	AATTAGGACTAGCCATCCAC	**BM1 TNF**	TTATCTCTCAGCTCCACG
		**FM1 TGFb1**	AACCCACAACGAAATCTATG
		**BM1 TGFb1**	CTTTTAACTTGAGCCTCAGC
		**FM1 XCL1**	TACATTGTGGAAGGTGTAGG
		**BM1 XCL1**	TGGTGTAGGTCTTGATTCTG
		**FM1 YWHAZ**	AACCTTGACATTGTGGACATC
		**BM1 YWHAZ**	AAAACTATTTGTGGGACAGC

Supplementary Figure Legends

Figure S1:

The indicated cell lines were irradiated with 10 Gy ionising radiation and after 4hrs fixed and immunostained for RAD51 and γH2AX. The absence of RAD51 foci in cells with γH2AX foci after irradiation indicated homologous recombination defect (HRD).

Figure S2:

IncuCyte assay analysis of total cell number and % cell death measured using Sytox Green uptake for indicated ovarian cancer cells. Cells were treated with; Control (vehicle), CHK1i (1 μM SRA737), LDHU (low-dose HU, 0.2 mM), HDHU (high-dose HU, 2 mM) or the indicated combinations. The data are the mean and SD of 6-30 determinations. These are representative of 2-3 experiments.

Figure S3:

IncuCyte assay analysis of total cell number and % cell death measured using Sytox Green uptake for indicated ovarian cancer cells. Cells were treated with; control (vehicle) or CHK1i (1 μM SRA737) + LDHU (low-dose HU, 0.2 mM). The data are the mean and SD of 6-30 determinations. These are representative of 2-3 experiments. These data were used to produce the % viable cell data in Figure 1C.

Figure S4:

CHK1i combination induces DNA damage and replication stress in ovarian cancer cells. Ovarian cancer cell lines and patient derived cells were treated with or without the CHK1i

combination (1 μM SRA737 + 0.2 mM HU) for 24 hours, followed by cell harvesting and Western blot analysis of the indicated markers. The band intensities relative to the control for each cell line are shown for each band. The upper and lower numbers are for the respective treated bands for RPA2. This is representative of three individual experiments.

Figure S5:

Ascites volume and tumour weights from ID8 p53-/- tumour bearing mice either treated with vehicle control or 50 mg/kg SRA737 alone or in combination with our LDHU protocol. Mice were harvested at ethical endpoint for the controls and the ascites volume and tumour weights measured. The data are mean and SD. Statistical analysis was performed using One-Way ANOVA test. * $p<0.05$, ** $p<0.01$.

Figure S6:

A) Gating strategies for experiments shown in Figure 6A,B and Supplementary Figure S6A, and **B)** Figure 6C,D.

Figure S7:

A) ID8 $p53^{WT}$ tumours were established in immunocompetent mice with treatment commenced day 21. Omental tumour samples were processed and stained with immune cell markers. The relative abundance of the indicated major immune cell types in either the myeloid (MDSCs, macrophages, DCs, NK cells, B cells), or lymphoid cell compartments ($CD4^+$ and $CD8^+$ T cells, Tregs) were assessed using a similar gating strategy as in Supplementary Figure S5A. Statistical analysis using two-way ANOVA and Uncorrected Fisher's LSD.

B) Depletion of $CD8^+$ T cell. Representative plots of FACS analysis to confirm $CD8^+$ T cell depletion in blood at the CHK1i+LDHU treatment endpoint. Bars represent the mean ± SD. Statistical analysis was performed by One-way ANOVA. The level of $CD8^+$ T cells in the peripheral blood of mice harvested at the endpoint of the experiment shown in Figure 6E.

1. Emoto M, Oshima K, Ishiguro M, Iwasaki H, Kawarabayashi T, Kikuchi M. Establishment and Characterization of a Serous Papillary Adenocarcinoma Cell Line of the Human Ovary in a Serum-free Culture. *Pathology - Research and Practice* 1999, **195**(4)**:** 238-243.

2. Hamilton TC, Young RC, McKoy WM, Grotzinger KR, Green JA, Chu EW*, et al.* Characterization of a human ovarian carcinoma cell line (NIH:OVCAR-3) with androgen and estrogen receptors. *Cancer research* 1983, **43**(11)**:** 5379-5389.

3. Wolf CR, Hayward IP, Lawrie SS, Buckton K, McIntyre MA, Adams DJ*, et al.* Cellular heterogeneity and drug resistance in two ovarian adenocarcinoma cell lines derived from a single patient. *International Journal of Cancer* 1987, **39**(6)**:** 695-702.

Supplementary Figure S1

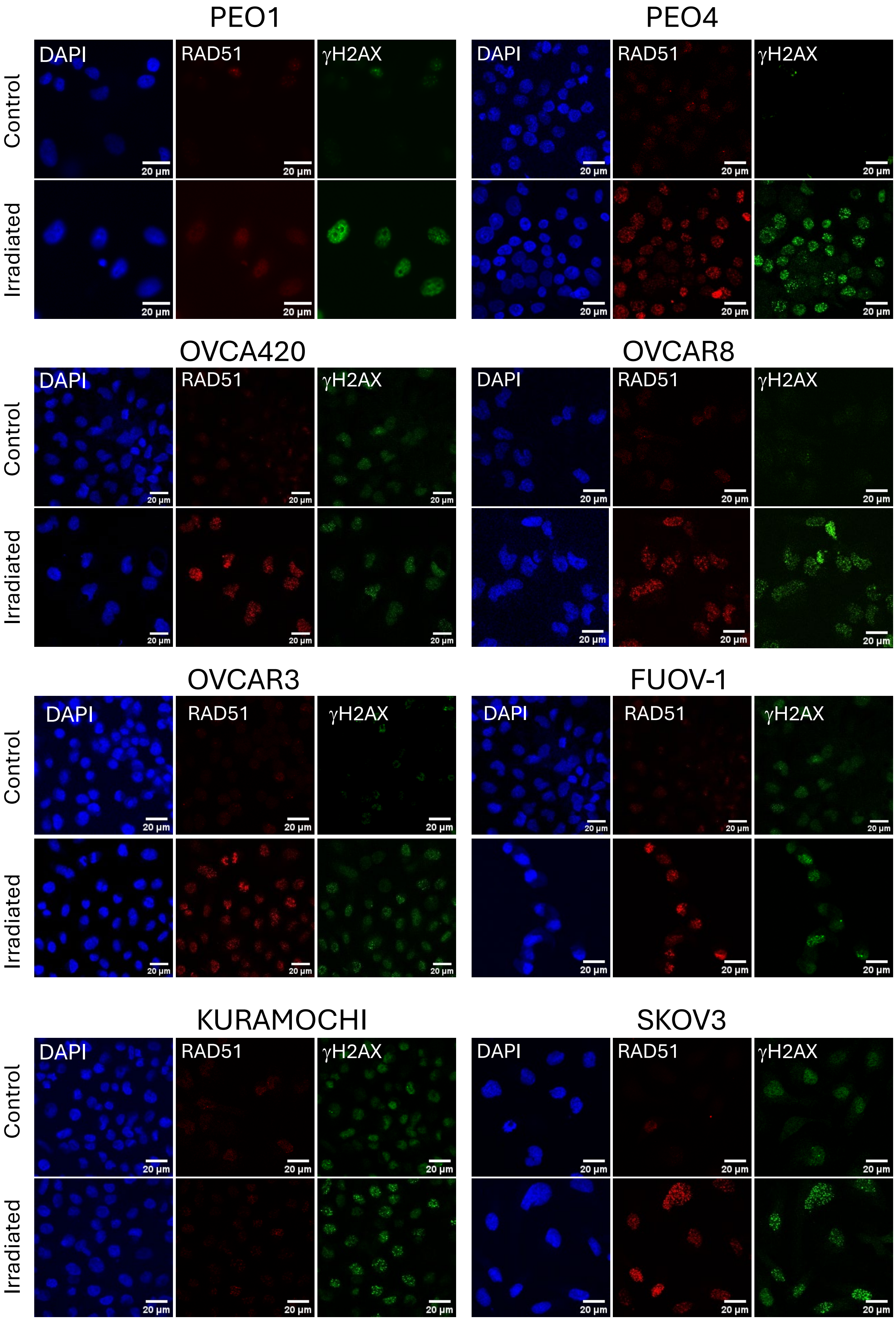

Supplementary Figure S1

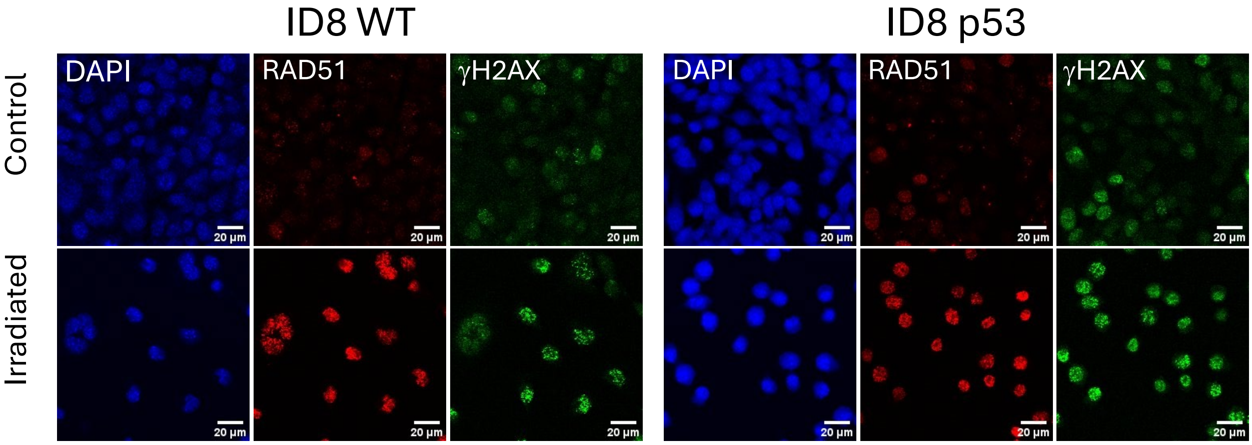

Supplementary Figure S2

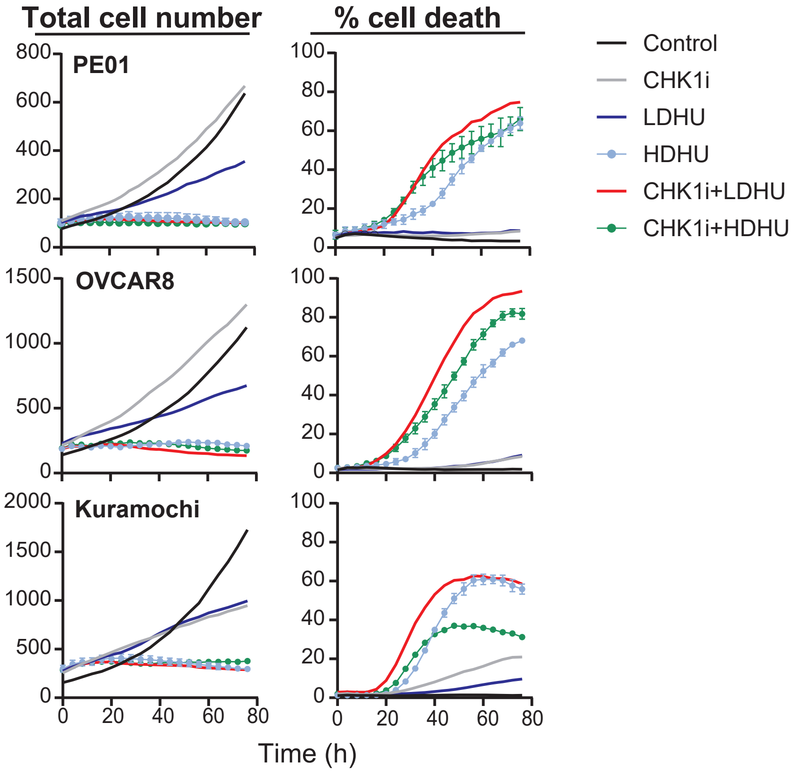

Supplementary Figure S3

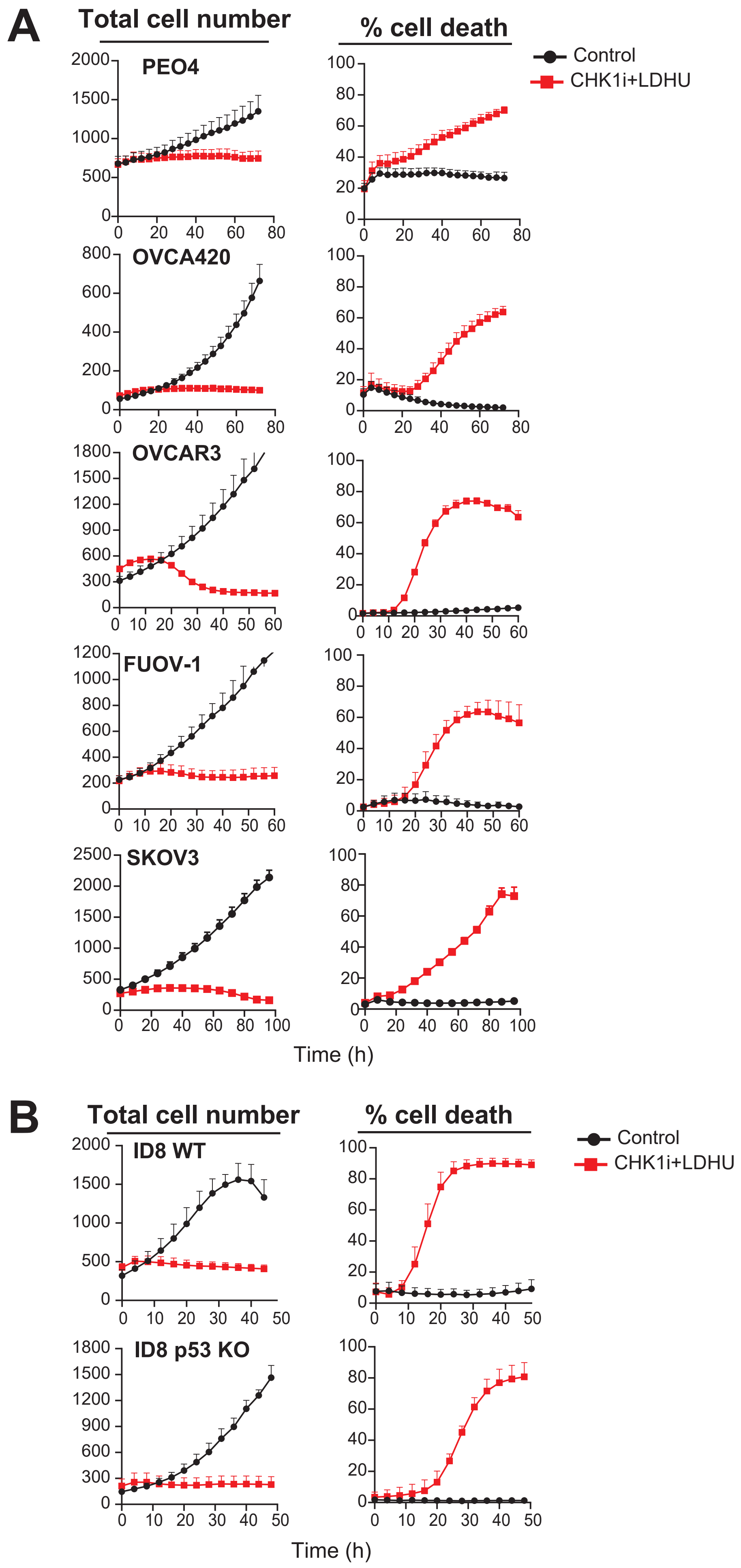

Supplementary Figure S4

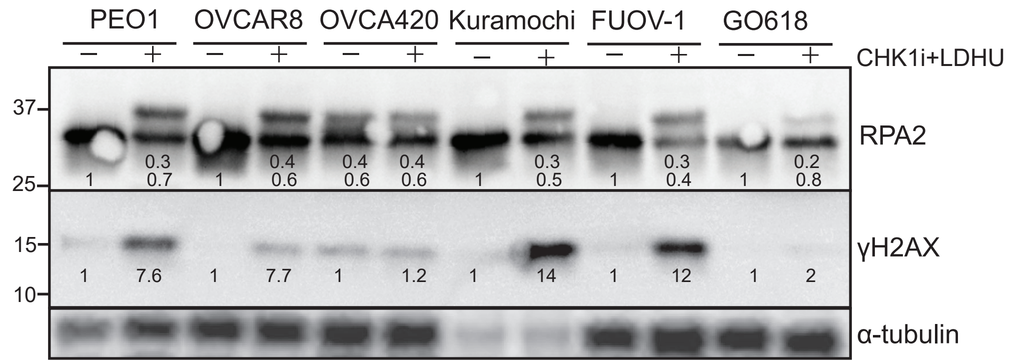

Supplementary Figure S5

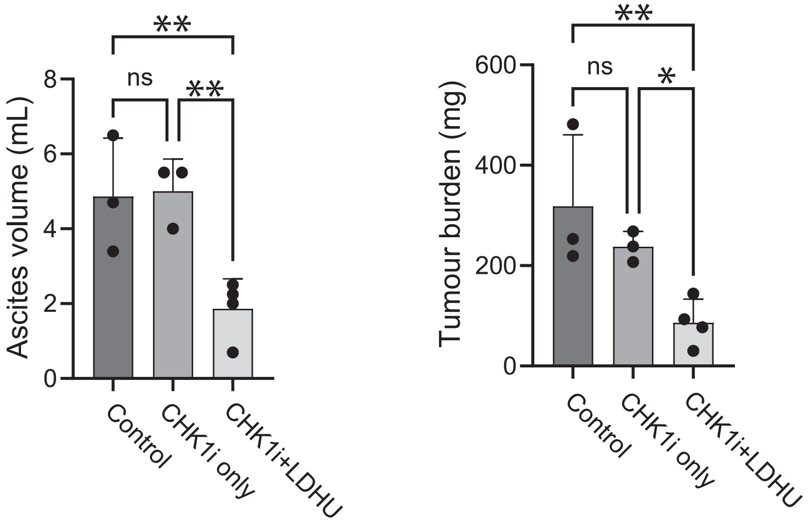

Supplementary Figure S6

A

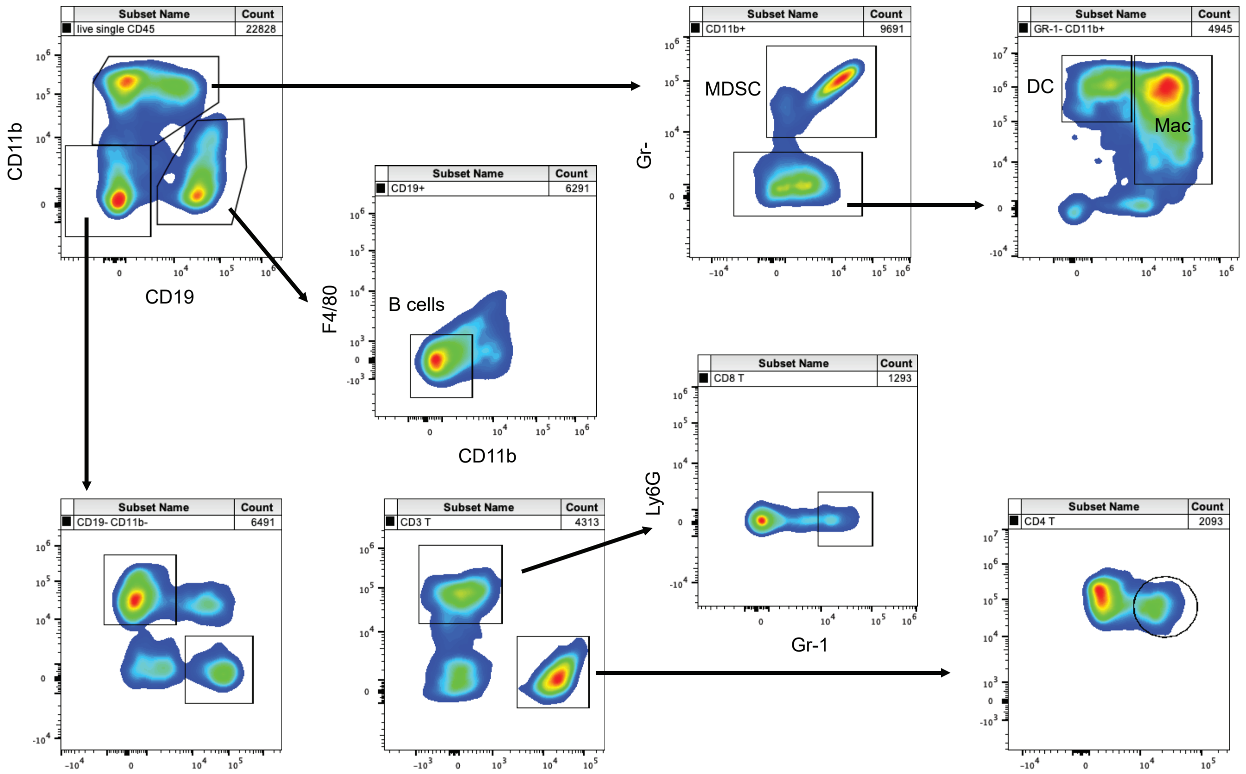

B

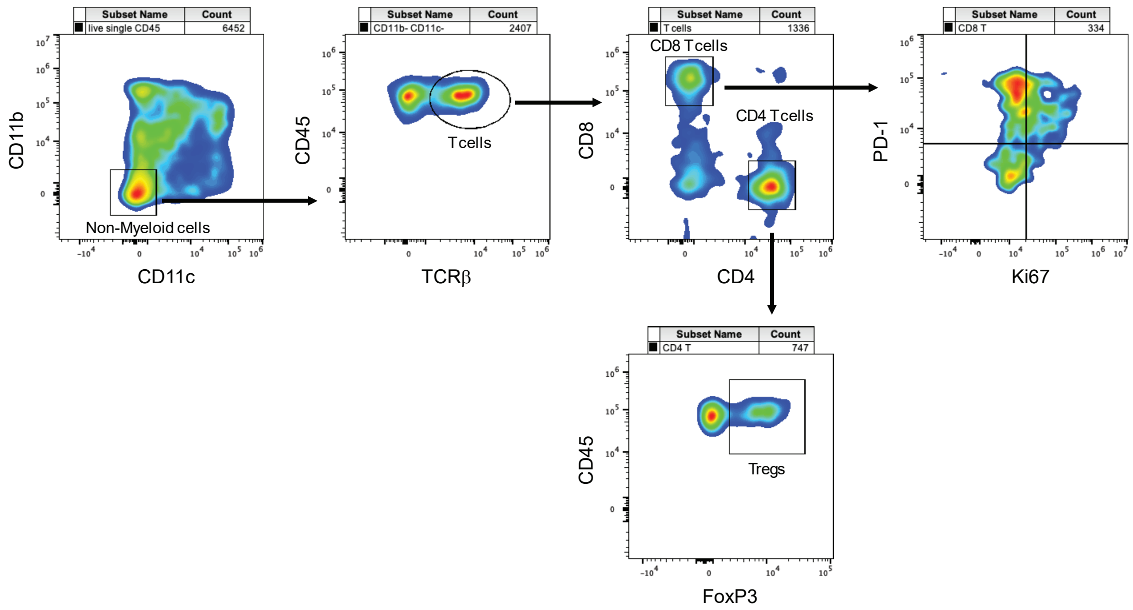

Supplementary Figure S7

A

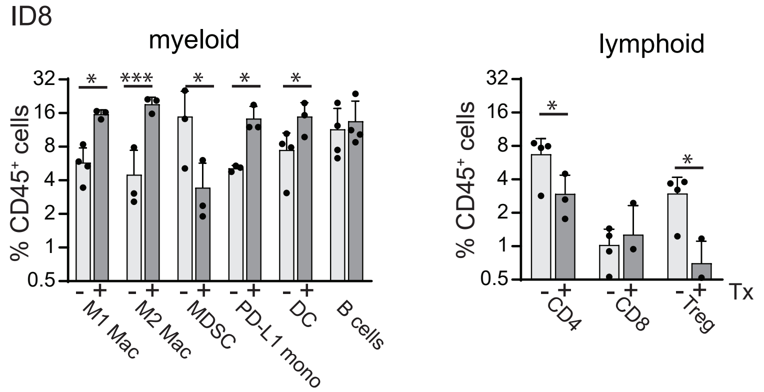

B

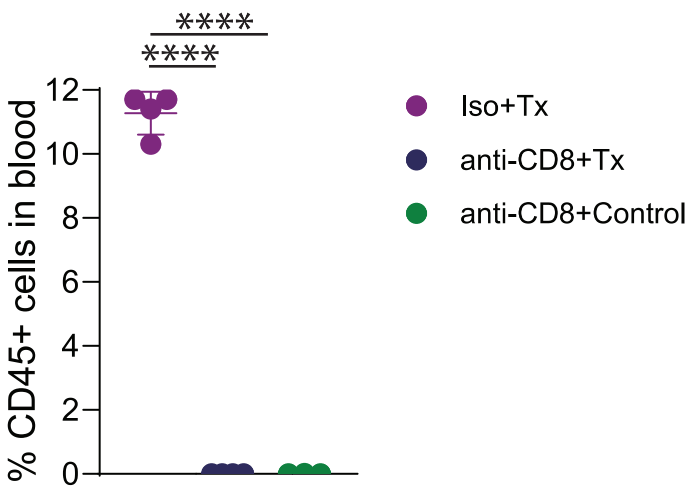

Supplement: Supplementary file 1 — Supplementary Information [file 41416_2026_3416_MOESM1_ESM.pdf]
